# ANGPTL4, IL-6 and TNF-α as regulators of lipid metabolism during a marathon run

**DOI:** 10.1038/s41598-022-17439-x

**Published:** 2022-11-19

**Authors:** Monika Górecka, Krzysztof Krzemiński, Tomasz Mikulski, Andrzej Wojciech Ziemba

**Affiliations:** Clinical and Research Department of Applied Physiology, Mossakowski Medical Research Institute, Polish Academy of Sciences, 5 Pawińskiego Street, 02-106 Warsaw, Poland

**Keywords:** Fat metabolism, Fatty acids, Interleukins, Tumour-necrosis factors

## Abstract

The aim of the study was to reveal whether marathon running influences regulators of lipid metabolism i.e. angiopoietin-like protein 4 (ANGPTL4), interleukin 6 (IL-6) and tumor necrosis factor-α (TNF-α). Plasma concentration of ANGPTL4, IL-6, TNF-α and lipids were determined in samples collected from 11 male runners before the marathon, immediately after the run and at 90 min of recovery. Plasma ANGPTL4 increased during exercise from 55.5 ± 13.4 to 78.1 ± 15.0 ng/ml (P < 0.001). This was accompanied by a significant increase in IL-6, TNF-α, free fatty acids (FFA) and glycerol (Gly) and a decrease in triacylglycerols (TG). After 90 min of recovery ANGPTL4 and TG did not differ from the exercise values, while plasma IL-6, TNF-α, FFA and Gly concentration were significantly lower. The exercise-induced increase in plasma concentration of ANGPTL4 correlated positively with the rise in plasma IL-6, TNF-α, FFA and Gly and negatively with the duration of the run. The increase in plasma IL-6 and TNF-α correlated positively with the rise in Gly. Summarizing, marathon running induced an increase in plasma ANGPTL4 and the value was higher in faster runners. The increase in plasma FFA, IL-6 and TNF-α concentration during a marathon run may be involved in plasma ANGPTL4 release, which could be a compensatory mechanism against FFA-induced lipotoxicity and oxidative stress. All of the analyzed cytokines may stimulate lipolysis during exercise.

## Introduction

The energy substrates for prolonged exercise, such as a marathon run, involve both carbohydrates and lipids. The highest oxidation of fatty acids occurs at 63% of maximal oxygen uptake (VO_2_max), which closely reflects the intensity of a marathon run in slower, recreational runners, while faster runners perform at higher intensities (70–75% of VO_2_max) and shift more towards carbohydrates^1,2^. The exercise duration is also important: after 90–120 min of exercise the contribution of fatty acids oxidation towards covering energy demand increases significantly^3^. Practically, lipid metabolism is of great importance for marathon runners in order to avoid the “hitting the wall” phenomenon and conditioning the aerobic capacity is one of the key factors in the adaptation to endurance training^4,5^. The source of fatty acids oxidized during exercise originates from triacylglycerols (TG) stored in skeletal muscles and adipose tissue, as well as in chylomicrons and very low density lipoproteins (VLDL) circulating in blood^6–8^. Lipolysis of intracellular TG is catalysed successively by three lipases: adipose triglyceride lipase, hormone-sensitive lipase and monoacyglycerol lipase^7,8^. Hydrolysis of plasma TG-rich lipoproteins takes place at the luminal surface of capillary endothelium with the participation of lipoprotein lipase (LPL)^9,10^. It was demonstrated that baseline LPL activity of both adipose tissue and skeletal muscles was significantly higher in long-distance runners than in untrained men^11,12^.

Besides other hormones, neurotransmitters and cytokines, the molecules involved in glucose and lipid metabolism during various forms of physical activity, as well as in inflammatory processes, include angiopoietin-like protein 4 (ANGPTL4), interleukin 6 (IL-6) and tumor necrosis factor-α (TNF-α)^13–15^. All of them stimulate intracellular adipocyte lipolysis, thus increasing the plasma level of free fatty acids (FFA) and glycerol (Gly)^16–18^. Moreover, ANGPTL4 catalyses the inactivation of the LPL monomers and locally reduces the uptake of TG-derived fatty acids into the tissue^19–21^. Makoveichuk et al. found that ANGPTL4 was a crucial mediator of the inhibitory effect of TNF-α on LPL activity in adipose tissue^22^. Interestingly, experimental studies have shown that TNF-α, unlike IL-6, exerts no effect on the oxidation of fatty acids in muscles, but increases its incorporation into diacylglycerols^23^.

It was demonstrated that the expression of ANGPTL4 was highly stimulated by FFA which are agonists for peroxisome proliferator-activated receptors (PPARs) in myocytes, cardiomyocytes, macrophages (PPAR-δ), adipocytes (PPARγ) and hepatocytes (PPARα)^10,19,24^. Several studies showed that strenuous exercise associated with muscle soreness markedly induced the expression of ANGPTL4 in adipose tissue, skeletal muscles and the liver^25–27^. Contracting skeletal muscles were also found to be the main sources of circulating IL-6 and TNF-α during exercise, while in the recovery period the molecules were mainly released from adipose tissue^28–30^. The other factors that can stimulate the production and secretion of ANGPTL4, IL-6 and TNF-α include: low glucose availability, inflammation, oxidative stress, catecholamines, hypoxia and hyperthermia^13,24,28,31^.

It has been proven that prolonged strenuous exercise triggers an omnidirectional inflammatory response which involves the release of both pro-inflammatory (IL-1, IL-8, TNF-α, IFN-γ, VEGF) and anti-inflammatory (IL-2, IL-4, IL-10, IL-13) plasma cytokines^32^. The balance between pro- and anti-inflammatory cytokines release is critical for prevention of excessive inflammation and for promotion of tissue repair process. The major role in the immune response is played by toll-like receptors (trans-membrane proteins, TLR) that are widely distributed in both the immune and other body cells such as myocytes, adipocytes and hepatocytes^33^. Some studies reported that ANGPTL4 was involved in acute inflammatory response and showed a potentially anti-inflammatory activity^34,35^. A marked increase in plasma concentration of IL-1 receptor antagonist, IL-6, IL-8, IL-10 and TNF-α was observed in endurance-trained men immediately after the marathon completion^36,37^. To the best of our knowledge, to date, no studies have been conducted to assess the effect of a marathon run on plasma ANGPTL4 in healthy, endurance-trained men.

The purpose of the present study was to investigate the effect of a marathon run on plasma ANGPTL4 and to find out whether the exercise-induced changes in plasma ANGPTL4 are related to those of plasma IL-6, TNF-α and lipids concentration.

## Materials and methods

### Subjects

The study included eleven well-trained endurance runners with three years’ experience in physical training and endurance events. Table 1 presents the general characteristics of the subjects. None of the subjects had serum lipid parameters outside the normal range. The whole research was performed in accordance with relevant regulations, including the Declaration of Helsinki. The procedure was approved by the Local Ethics Committee at Medical University of Warsaw, permission number KB/73/A/2014. All subjects signed the informed consent statement and underwent a medical examination prior to being included in a study.

**Table 1:** Subject characteristics (*n* = 11).

Parameters	Mean ± SEM
Age (years)	33.7 ± 1.2
Body height (cm)	177.2 ± 1.9
Body mass (kg)	75.0 ± 2.0
Body mass index (kg/m^2^)	24.0 ± 0.8
Maximal oxygen consumption (ml/kg/min)	54.8 ± 1.2
Running history (years)	3.0 ± 0.6
Training volume (km/week)	60.4 ± 4.5
Training time (h/week)	5.6 ± 0.6
Running time in present race (h)	4.1 ± 0.2
Running speed in present race (km/h)	10.6 ± 0.5
Average running intensity (%VO_2_max)	67.8 ± 1.4

### Experimental protocol

The subjects were recruited from marathoners who completed a street marathon of a distance of 42.2 km (Warsaw Marathon). The run was held in September when the air temperature typically oscillates between 13 and 16 °C with 55–60% humidity and 1020–1021 hPa atmospheric pressure. The race started at 9:00 a.m., and the runners had to reach the finish line within 7 h. Our subjects’ individual finishing times ranged from 3.17 to 5.71 h. However, the average running speeds measured at 10, 21, 30 and 42.2 km were not significantly different from each other and amounted to 10.6 ± 0.5; 11.1 ± 0.5; 10.8 ± 0.6; and 10.2 ± 0.6 km/h, respectively. The subjects ran at the estimated mean intensity of 67.8 ± 1.4% of VO_2_peak. There were 15 service points every 2–3 km along the marathon route with water, isotonic drinks and high-carbohydrate snacks (bananas, lump sugar, drops, carbohydrate gels). The analysis of the runners’ reports revealed a mean intra-marathon energy intake of 848.4 ± 62.7 kcal and fluid intake of 2.79 ± 0.16 l. The mean hourly intake of carbohydrates was recorded at 51.9 ± 2.2 g/h and was in accordance with the recommendation of the American College of Sports Medicine to maintain oxidation of carbohydrates and delay fatigue^38^.

One week before the run, the subjects underwent the incremental, graded exercise test on a T2000 treadmill (GE, USA) until volitional exhaustion to determine their peak oxygen uptake. The treadmill speed started at 6 km/h and was increased by 2 km/h every 3 min. Pulmonary ventilation, O_2_ uptake, CO_2_ production were recorded using the V_max_ 29 system (SensorMedics, USA).

On the day of the marathon, after a 30-min pre-run resting period, immediately after the run and at the 90 min of recovery blood samples were taken from the antecubital vein to determine the plasma concentration of ANGPTL4, IL-6, TNF-α, TG, FFA, Gly, total cholesterol (TC), high-density lipoprotein-cholesterol (HDL-C), low-density lipoprotein-cholesterol (LDL-C) and glucose. Blood samples were collected in EDTA containing tubes and centrifuged at 1700×*g* for 15 min at 4 °C. Plasma samples were frozen at − 20 °C and then transported and stored at − 80 °C until assay. Whole-blood samples were used to measure lactate (LA) concentration and the hematocrit. The 90-min recovery period was selected based on the data from the available literature^39,40^.

### Blood sample analysis

The plasma ANGPTL4 concentration was measured by enzyme-linked immunosorbent assay using DuoSet ELISA Development kit (R&D Systems, USA) that recognized full-length ANGPTL4 in human plasma^41^. The intra-assay coefficient of variation of this method was 7.6% ± 0.7 (n = 70), while inter-assay coefficient of variation was 16.8% ± 1.8 (n = 33). High-sensitivity ELISA kits (Quantikine HS ELISA Human Immunoassay) provided by R&D Systems (USA) were used for the pre-run blood samples for IL-6 and TNF-α, because these cytokines exist at very low levels in peripheral blood. The intra-assay coefficient of variation of these methods for IL-6 and TNF-α equaled 4.1%, and 2.0%, respectively. Quantitative sandwich ELISA kits (R&D Systems, USA) were used for the post-run and recovery blood samples for IL-6 and TNF-α. The intra-assay coefficients of variation of these methods for IL-6 and TNF-α was 2.6%. The plasma FFA concentration was measured using standard enzymatic colorimetric assay (ACS-ACOD Method, Wako Chemicals GmbH, Germany). The intra-assay coefficient of variation was 1.5%. The plasma concentration of TG, Gly, TC, LDL-C and HDL-C was determined by enzymatic methods using manual diagnostic kits (Randox Laboratories Limited, United Kingdom). The intra-assay coefficients of variation for these assays did not exceed 6.4%. Plasma glucose concentration was measured enzymatically using Glucose Oxidase Reagent Set (Pointe Scientific Inc., USA). Lactate concentration was determined using strip analyzer Lactate Scout (EKF Diagnostics, Germany). Hematocrit was measured in duplicate. Whole blood samples (~ 9 μl) were transferred to heparinized microcapillary tubes and analyzed by an automated system following microcentrifugation. Hematocrit was used to calculate percent changes in plasma volume. The percentage changes in plasma volume were calculated using Van Beaumont's equation^42^.

### Statistical analysis

The statistical analysis was performed according to the previously presented scheme^40^. The data are presented as means with standard errors (SEM). The normality of variables distribution was checked with the Shapiro–Wilk test. The homogeneity of variances was tested with the Levene test. Normally distributed parameters were compared using one-way analysis of variance (ANOVA) for repeated measures. When the ANOVA revealed a statistically significant effect (P < 0.05), the Bonferroni test was used for “post hoc” comparisons. Variables with non-normal distribution (TNF-α, IL-6, FFA, Gly, LA, TC, LDL-C, TG/HDL-C), were compared using the Friedman ANOVA rank test and the Wilcoxon test with the Bonferroni correction for multiple comparisons (P < 0.02). Correlation coefficients were calculated between normally distributed variables (the exercise-induced increases, deltas) using the Pearson’s linear regression analysis and Spearman’s correlations for the non-normally distributed recovery data (P < 0.05 was accepted as the level of significance). For calculations, the Statistica 6 software was used (Statsoft Inc., USA).

## Results

### ANGPTL4, TNF-α, IL-6, FFA, Gly, TG, TC, HDL-C and LDL-C

**Figure 1:**
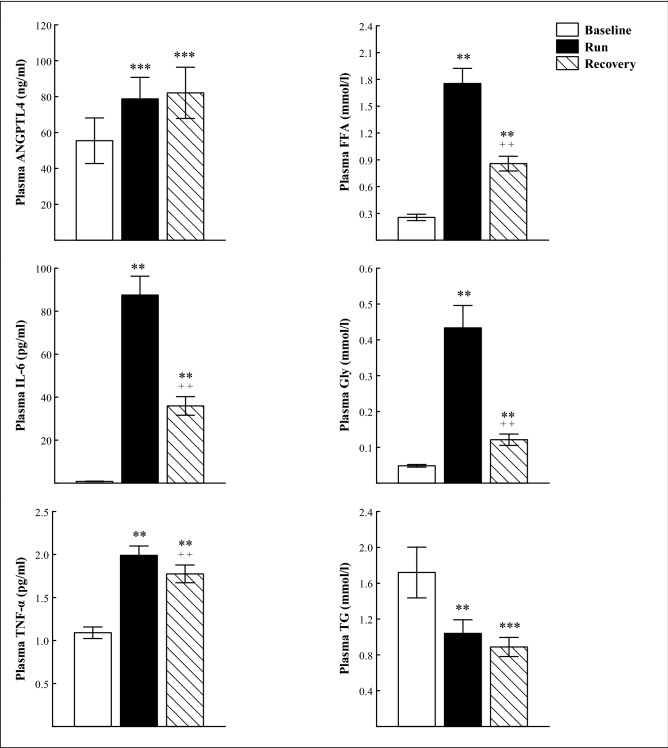
The plasma concentration of angiopoietin-like protein 4 (ANGPTL4), interleukin 6 (IL-6), tumor necrosis factor-α (TNF-α), free fatty acids (FFA), glycerol (Gly) and triacylglycerols (TG) before (Baseline), immediately after marathon run (Run) and at the 90 min of recovery (Recovery). The values are mean ± SEM (*n* = 11). Asterisks denote significant differences from the resting values: **P < 0.01, ***P < 0.001. Crosses denote significant differences from the exercise values: ^++^P < 0.01.

**Figure 2:**
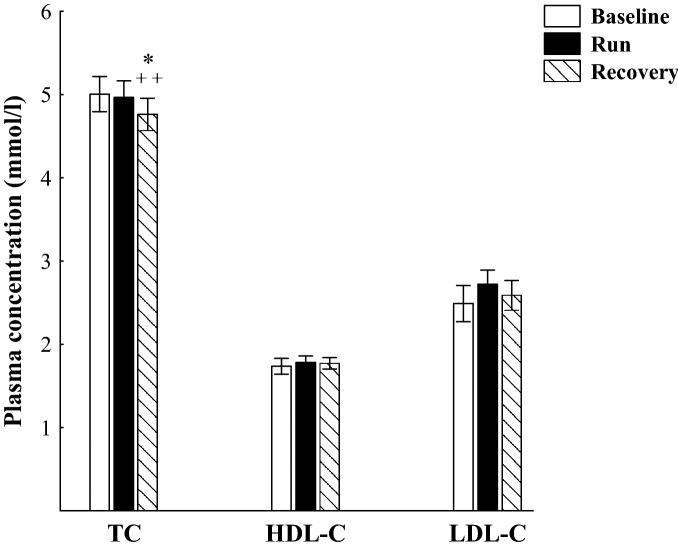
The plasma concentration of total cholesterol (TC), high-density lipoprotein-cholesterol (HDL-C) and low-density lipoprotein-cholesterol (LDL-C) before (Baseline), immediately after marathon run (Run) and at the 90 min of recovery (Recovery). The values are mean ± SEM (*n* = 11). Asterisks denote significant differences from the resting values: *P < 0.02. Crosses denote significant differences from the exercise values: ^++^P < 0.01.

Immediately after the marathon run, a significant increase was found in plasma concentration of ANGPTL4 (P < 0.001), TNF-α, IL-6, FFA and Gly (P < 0.01), whereas TG concentration was observed to decrease (P < 0.01; Fig. 1). Directly after the run, plasma concentration of TC and its fractions (HDL-C and LDL-C) did not differ from the baseline values (Fig. 2), but molar ratio of TG/HDL-C was significantly reduced (P < 0.01; Table 2). Plasma ANGPTL4 concentration recorded 90 min after the run was similar to that obtained immediately after the exercise, whereas plasma concentration of TNF-α and IL-6, as well as FFA and Gly, were significantly decreased compared to the exercise values (P < 0.01), but they were still higher than the baseline values (P < 0.01; Fig. 1). During the recovery, plasma TG concentration remained unaffected but it was significantly lower than before the run (P < 0.001; Fig. 1). At the 90 min of recovery plasma TC concentration was markedly decreased compared to the exercise (P < 0.01) and baseline (P < 0.02) values (Fig. 2), whereas the average molar ratios of TC/HDL-C and TG/HDL-C were significantly reduced compared to the baseline values (Table 2).

**Table 2:** The molar ratios of total cholesterol to high-density lipoprotein-cholesterol (TC/HDL-C) and triacylglycerols to high-density lipoprotein-cholesterol (TG/HDL-C) before (Baseline), immediately after marathon (Run) and at 90 min of recovery (Recovery) (*n* = 11). The values are means ± SEM. Asterisks denote significant differences from the resting values: **P < 0.01.

Parameters	Baseline	Run	Recovery
TC/HDL-C	2.95 ± 0.17	2.83 ± 0.14	2.72 ± 0.13 **
TG/HDL-C	1.10 ± 0.25	0.61 ± 0.11 **	0.52 ± 0.07 **

**Figure 3:**
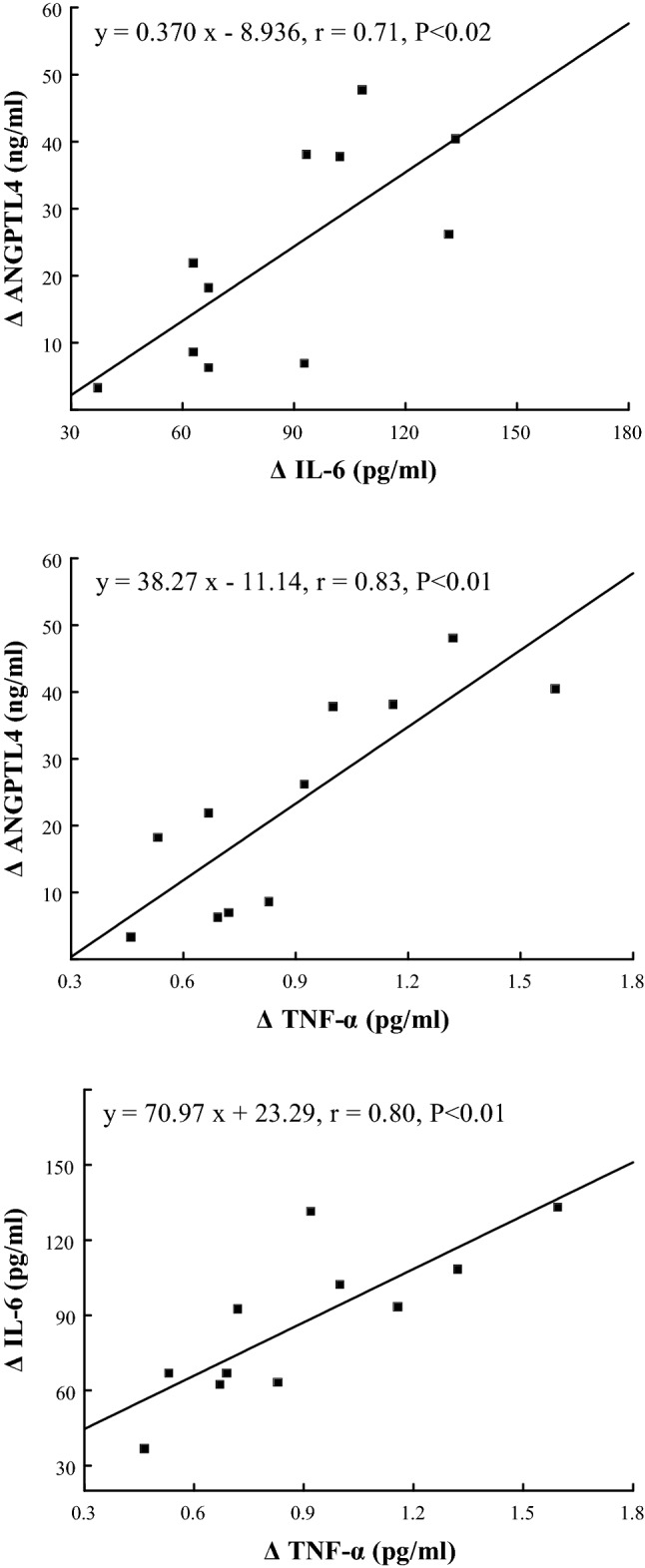
The relationships between exercise-induced changes in plasma concentration of angiopoietin-like protein 4 (ANGPTL4) and those of plasma interleukin 6 (IL-6), tumor necrosis factor-α (TNF-α) as well as between exercise-induced changes in plasma concentration of IL-6 and TNF-α.

**Figure 4:**
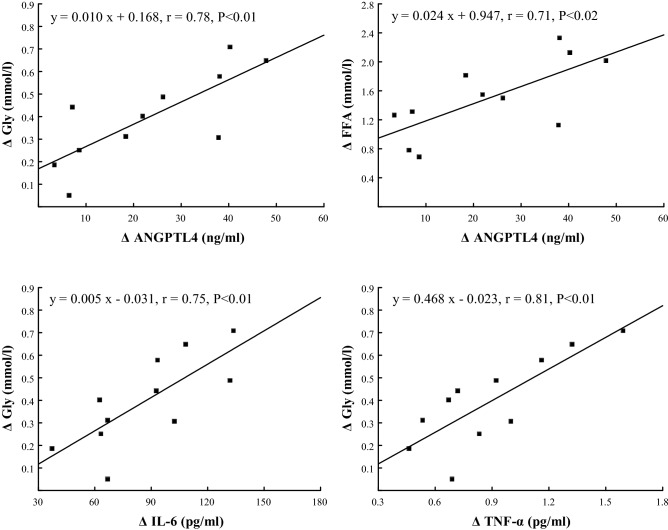
The relationships between exercise-induced changes in plasma concentration of free fatty acids (FFA), glycerol (Gly) and those of plasma angiopoietin-like protein 4 (ANGPTL4), interleukin 6 (IL-6) and tumor necrosis factor-α (TNF-α).

**Figure 5:**
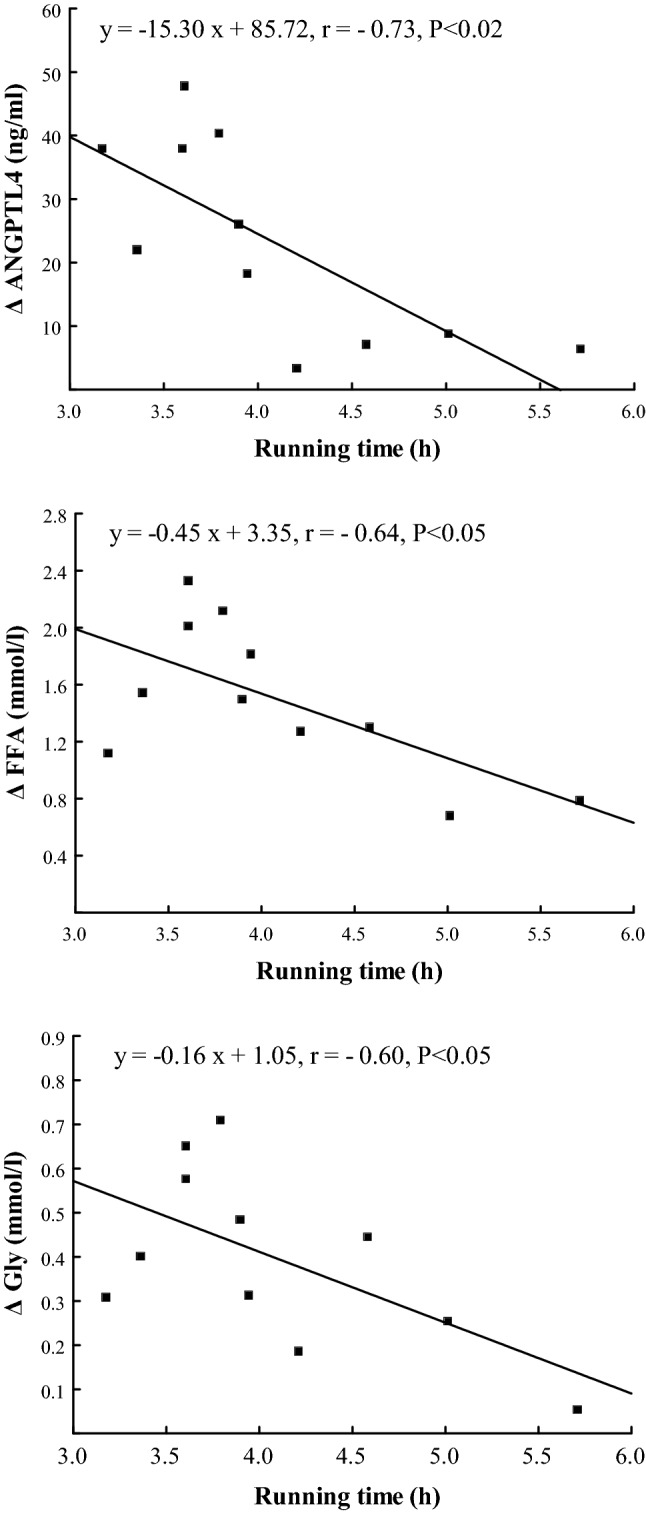
The relationships between exercise-induced changes in plasma concentration of angiopoietin-like protein 4 (ANGPTL4), free fatty acids (FFA) and glycerol (Gly) and running times obtained by marathoners.

The exercise-induced increase in plasma concentration of ANGPTL4 correlated positively with the rise in plasma IL-6 (r = 0.71, P < 0.02), TNF-α (r = 0.83, P < 0.01), FFA (r = 0.71, P < 0.02) and Gly (r = 0.78, P < 0.01) (Figs. 3 and 4), and correlated negatively with the duration of the run (r = − 0.73, P < 0.02) (Fig. 5). The exercise-induced changes in plasma IL-6 concentration correlated with the fluctuation of plasma TNF-α concentration (r = 0.80, P < 0.01) (Fig. 3). The increases in plasma IL-6 and TNF-α correlated positively with those of Gly (r = 0.75 and r = 0.81, respectively, both P < 0.01) (Fig. 4). The exercise-induced increases in plasma FFA and Gly correlated negatively with individual running times (r = − 0.64, r = − 0.60, respectively, both P < 0.05) (Fig. 5). The positive correlation between plasma concentration of IL-6 and TNF-α was found at the 90th min of recovery (r = 0.73, P < 0.05).

### Glucose, LA, Hematocrit

Plasma glucose concentration did not differ significantly from the baseline level. Plasma LA concentration was significantly increased immediately after the run (a rise from 1.66 ± 0.16 mmol/l to 3.93 ± 0.40 mmol/l was recorded) (P < 0.01). The exercise-induced changes in plasma LA correlated negatively with individual running times (r = − 0.68, P < 0.03). No significant changes were found in hematocrit.

## Discussion

The new finding of the present study is that the marathon run induced a significant increase in the plasma ANGPTL4 concentration in healthy, endurance-trained, young men, which was accompanied by a significant increase in plasma concentration of IL-6, TNF-α, FFA and Gly. Furthermore, significant positive correlations were found between exercise-induced increases in plasma ANGPL4 and those of plasma IL-6, TNF-α, FFA and Gly. The positive relationship between exercise-induced changes in plasma ANGPTL4 and those of plasma FFA shown in this study confirmed the data obtained in our previous investigation performed in healthy young men during a mountain ultra-marathon run^40^. We argued then that the elevated level of ANGPTL4 increased plasma FFA and the raised FFA would boost the release of ANGPTL4. Many authors have reported that ANGPTL4 may, independently of other factors such as catecholamines, cortisol, glucagon, growth hormone, IL-6 or TNF-α, stimulate adipocyte tissue lipolysis leading to the elevation of plasma FFA level^18,43^. It has also been shown that ANGPTL4 simultaneously raises plasma TG level by suppressing lipoprotein lipase (LPL)-mediated clearance of plasma TG-rich lipoproteins^31,44^. The inhibition of LPL by ANGPTL4 occurs before GPIHBP1 (glycosylphosphatidylinositol-anchored high density lipoprotein-binding protein 1) interaction with LPL and its transport to the luminal site of the capillary endothelium^19,21^.

In the present study the exercise-induced increase in plasma concentration of ANGPTL4, FFA and Gly was observed to reach higher values in faster runners (who deplete carbohydrate stores more rapidly) and the rise correlated negatively with the running time. Moro et al. have found that the rate of adipose tissue lipolysis and plasma FFA concentration depend on the availability of glycogen in working muscles^45^. A greater lipolytic response could not be explained solely by significant changes in lipolytic stimuli, such as increased catecholamines, atrial natriuretic peptide, growth hormone, cortisol and IL-6 or a decrease in circulating insulin during moderate intensity exercise. The emerging evidence indicates that contracting skeletal muscles can release specific substances called exerkines which enable remote signaling with adipose tissue^46^. Therefore, the present results may suggest that ANGPTL4 could play a role of an exerkine that is produced in case of reduced glycogen availability in working skeletal muscles or in the liver to promote lipolysis in adipose tissue.

The data obtained by Kersten et al. suggest that the effect of endurance exercise, severe energy restriction and fasting on plasma ANGPTL4 may be mediated by an elevated plasma FFA via PPARα and PPARβ/δ^24^. Robciuc et al. demonstrated that the activation of PPARδ/retinoic X receptor (RXR) upregulated ANGPTL4 gene expression causing the inhibition of LPL activity and LPL-dependent FFA uptake in myotubes^19^. The activation of the FFA-PPARδ/RXR-ANGPTL4 axis functions as a negative feedback mechanism that may serve to protect the skeletal muscle fibers from lipid overload and the related oxidative stress^19,34^. Interestingly, Staiger et al. found that the induction of the ANGPTL4 gene expression in myotubes was independent of the degree of saturation and the length of the FFA^47^. The PPAR/RXR heterodimer can be activated by FFA produced locally via LPL-mediated TG hydrolysis of VLDL and chylomicrons, as well as by FFA derived from adipocyte lipolysis. Strong evidence suggests that 5′AMP-activated protein kinase (AMPK) activation, as would occur in exercising muscle, counteracted FFA induction of ANGPTL4^25^.

The exercise-induced increase in plasma ANGPTL4 observed in the present study correlated positively with the changes in plasma TNF-α and IL-6. Several studies have reported increased plasma IL-6 and TNF-α levels in endurance-trained men immediately following a marathon run^37,48^. Cho et al. found that TNF-α released from infiltrated macrophages stimulated ANGPTL4 expression in mesenchymal stem cells by retinoic acid receptor-related orphan receptor which binds to ANGPTL4 gene promoter^35^. It was also showed that human recombinant TNF-α increased plasma concentration of IL-6, which in turn inhibited TNF-α production^17,36^. TNF-α, unlike IL-6, had no impact on muscle fatty acid oxidation but it increased the release of free fatty acids from adipose tissue and their incorporation into diacylglycerol, which may be involved in the development of TNF-α-induced insulin resistance in skeletal muscles^23^*.* Bernecker et al. reported a twofold increase in TNF-α after a marathon run in addition to a nearly 100-fold increase in IL-6 values^37^. Several studies showed that IL-6 release from contracting muscle was regulated in response to exercise, being affected by the intensity and duration of exercise as well as by energy substrates availability and muscle mass involved^28,49^. The main stimulus for IL-6 synthesis and release during exercise is provided by muscle contraction, however, the transcription factors that are known to regulate IL-6 synthesis could also be activated by the decrease in muscle glycogen content and increased formation of reactive oxygen species^49^. Moreover, IL-6 produced in contracting skeletal muscles can act in an autocrine manner and stimulate muscle IL-6 synthesis^50^. Lipid turnover is enhanced by IL-6, stimulating fat oxidation and lipolysis in conjunction with catecholamines and cortisol^16,51^. IL-6 has also been reported to mobilize extracellular substrates and augment substrate delivery during exercise^52^. In several studies IL-6 was found to correlate positively with an increase in FFA which are an important energy substrate during strenuous endurance exercise^16,51^.

It has been reported that muscle and joint trauma results in the activation of circulating monocytes which, in turn, produce large quantities of pro-inflammatory IL-1, IL-6, and TNF-α^53^. Starkie et al. speculated that more than a 100-fold increase in plasma IL-6 concentration after a marathon run could be attributed to muscle damage^36^. The authors found that the elevated plasma IL-6 was accompanied by an increase in creatine kinase which is an indicator of muscle membrane damage. However, some studies have shown that the muscle damage-induced production of IL-6 is significantly lower than the production of IL-6 triggered by muscle contractions^54^. There is evidence that IL-6 and TNF-α are released from both damaged myofibers and infiltrating immune cells (fibro-adipogenic progenitors (FAP cells, muscle interstitial mesenchymal cells) and macrophages and neutrophils), which is required for muscle regeneration and repair^30,55,56^.

The present study also showed significant positive correlations between exercise-induced increases in plasma Gly and both IL-6 and TNF-α. This indicates that these cytokines may be involved in adipose tissue lipolysis and fatty acid mobilization during the marathon run. Interestingly, FFA have been suggested to induce chronic low-grade inflammation and activate the innate immune system. It was found that saturated FFA induced inflammation, while polyunsaturated FFA produced anti-inflammatory effect^57^. Hartung et al. noted a progressive increase in the ratio of unsaturated to saturated FFA during the marathon run^58^. Some authors suggested that local FFA efflux from adipocytes may be an important regulator of inflammation and macrophage recruitment to adipocyte tissue^59^. There is some evidence that saturated fatty acids serve as a ligand for TLR4, thereby inducing the inflammatory changes in both adipocytes and macrophages through the nuclear factor kappa B (NF-κB) activation^60^. NF-κB dimers activate the transcription of many κB-dependent genes, such as the genes of pro-inflammatory cytokines, including IL-6, TNF-α and IL-1β. Adipose tissue macrophages are a prominent source of these pro-inflammatory cytokines and together with adipocytes interact in a paracrine manner^60^. Polyunsaturated fatty acids, unlike saturated fatty acids, can reduce the synthesis of TNF-α and IL-6 by downregulating NF-κB. The anti-inflammatory effects of IL-6 have been shown to be related to a crosstalk between muscle tissue and adipose tissue.

## Conclusions

Marathon running induced an increase in plasma ANGPTL4 and the value was higher in faster runners. Increases in plasma FFA, IL-6 and TNF-α are probably involved in the release of ANGPTL4. Significant positive relationships between plasma ANGPTL4 and FFA may indicate that the elevated level of FFA stimulates ANGPTL4 release to circulation and the increased concentration of ANGPTL4 could increase FFA mobilization from adipose tissue. Enhanced ANGPTL4 secretion is a potential compensatory mechanism which prevents FFA-induced lipotoxicity and oxidative stress, especially in the tissues uninvolved in the exercise. ANGPTL4, IL-6 and TNF-α may, independently from catecholamines and other factors, stimulate adipose tissue lipolysis during prolonged exercise.

## Data Availability

All the data are available on request from the corresponding author.

## Abbreviations

ANGPTL4: Angiopoietin-like protein 4
FFA: Free fatty acids
Gly: Glycerol
HDL-C: High density lipoprotein-cholesterol
IL-6: Interleukin 6
LA: Lactate
LPL: Lipoprotein lipase
LDL-C: Low density lipoprotein-cholesterol
TC: Total cholesterol
TG: Triacylglycerols
TNF-α: Tumor necrosis factor-α
VLDL: Very low density lipoproteins
VO_2_ max: Maximal oxygen uptake
